# Gold Nanoparticles in Cancer Theranostics

**DOI:** 10.3389/fbioe.2021.647905

**Published:** 2021-04-13

**Authors:** Qinyue Gao, Jingjing Zhang, Jie Gao, Zhengyang Zhang, Haitao Zhu, Dongqing Wang

**Affiliations:** Department of Radiology, Affiliated Hospital of Jiangsu University, Zhenjiang, China

**Keywords:** optical property, nanomedicine, cancer theranostics, tumor microenvironment, gold nanoparticle

## Abstract

Conventional cancer treatments, such as surgical resection, radiotherapy, and chemotherapy, have achieved significant progress in cancer therapy. Nevertheless, some limitations (such as toxic side effects) are still existing for conventional therapies, which motivate efforts toward developing novel theranostic avenues. Owning many merits such as easy surface modification, unique optical properties, and high biocompatibility, gold nanoparticles (AuNPs and GNPs) have been engineered to serve as targeted delivery vehicles, molecular probes, sensors, and so on. Their small size and surface characteristics enable them to extravasate and access the tumor microenvironment (TME), which is a promising solution to realize highly effective treatments. Moreover, stimuli-responsive properties (respond to hypoxia and acidic pH) of nanoparticles to TME enable GNPs’ unrivaled control for effective transport of therapeutic cargos. In this review article, we primarily introduce the basic properties of GNPs, further discuss the recent progress in gold nanoparticles for cancer theranostics, with an additional concern about TME stimuli-responsive studies.

## Introduction

The researches of cancer treatments have been developing at an extremely rapid pace to support the personalized demands for better precision therapeutics. Currently, cancer treatment strategies for solid tumors include surgery, radiation therapy, chemotherapy, immunotherapy, and a combination of some or all of these mentioned approaches (Nicolson et al., 2020). Unsatisfactory antitumor performance and some severe side effects have always been a confusing problem in clinical treatments. The intrinsic limits of conventional cancer therapies prompt the increasing interests in the applying of nanotechnology in the diagnosis and therapy assessments for cancer, which fight cancer cells more accurately with fewer potential side effects. Nanotechnology (Wilhelm et al., 2019) as new treatment modalities, can greatly enhance the precise anticancer efficacy, showing promising potentials in clinical translations. Gold nanoparticles (GNPs) are of increasing interest because their excellent biocompatibility and physicochemical properties (Chen et al., 2016) can support multiple functions. Since the first scientific article attributing the red color to the colloidal nature of Au-based NPs reported by Faraday in 1857, AuNPs at present have been engineered as stable nanocargoes (Wang C. et al., 2018), metal catalysts (Vidal et al., 2018), organic photosensitizers (PS) (Li S. et al., 2019), or to directly produce ROS and heat under near-infrared light irradiation (Mangadlao et al., 2018), which promise biological friendly nanoparticles in the medical treatment and diagnostics.

**FIGURE 1:**
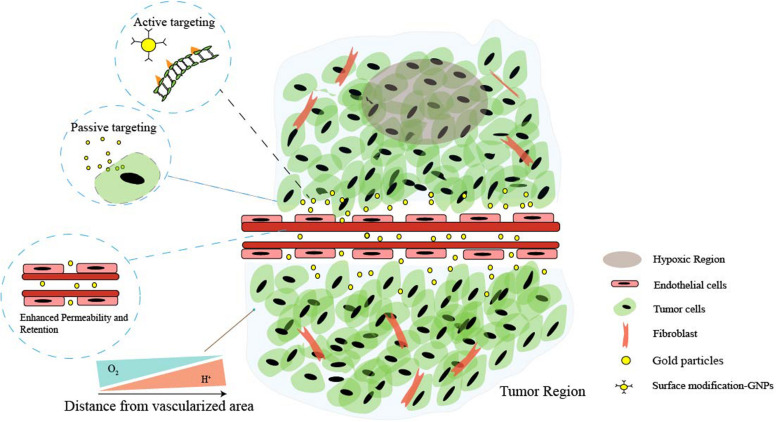
Schematic illustration of the main features of tumor microenvironment (TME). Cancer cells and non-malignant cells coexist in the extracellular matrix (ECM). Characteristics of the tumor microenvironment include acidity, hypoxia, hypertonicity, and recruitment of stromal and immune cells. Low perfusion of abnormal vessels typically limits both O_2_ delivery and removal of acidic waste products by oxidative metabolism and Warburg effect. Moreover, the gaps in the endothelial lining of cancer vessels are excessive (ranging from one to hundreds of nanometers), and nanoparticles can simply extravasate through these gaps, which is the enhanced permeability and retention (EPR) characteristic of TME. Additionally, the EPR effect enables nanoparticles to passively and preferentially accumulate and penetrate into the tumor, and through the surface affinity ligand modification, nanoparticles can actively target the specific receptors of cancer cells.

However, ideal antitumor functions of GNPs need to be favored by the tumor’s surrounding environment. The tumor microenvironment (TME) dictates tumor progression, regulates cancer cell behaviors, and persistently provides nutrition for cancer growth and metastasis (Figure 1). Several common features of TME, such as the vascular abnormalities, the extracellular matrix (hypertonicity, hypoxia, and pH), and the immune response of TME are quite different from normal tissues (Peng et al., 2019). These are the reasons that TME-triggered theranostic gold nanoparticles could offer promising perspectives to achieve tumor-specific detection and therapy (Table 2). In this review, we aim to illustrate the basic properties of GNPs and current researches on the development of GNPs including the interactions between nanomaterials and the TME.

## Properties of Gold Nanoparticles

### Localized Surface Plasmon Resonance

**FIGURE 2:**
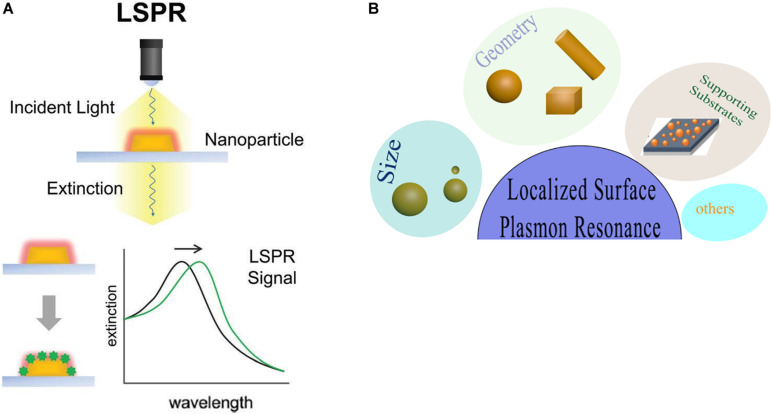
Local surface plasmon resonances (LSPR). **(A)** The measurement principles and spectral signatures of LSPR. Reprinted with permission from Jackman et al. (2017), Copyright (2017) American Chemical Society. **(B)** Physical effect of LSPR engineered by size, geometry, supporting substrates, and so on.

Gold nanoparticles spark broad interest due to their unique optical performance (Wilson et al., 2020). The external specific wavelength laser excites a collective and coherent oscillate of the conduction electrons near the gold nanoparticle’s surface, and this oscillate is in resonance with the incident light frequency leading to scattering peaks and spectral absorption as well as local field enhancements (Figure 2A), which is the important optical property of GNPs—localized surface plasmon resonance (LSPR) (Zhou et al., 2015; Ramos et al., 2018). Many studies have confirmed that LSPR of GNPs is exquisitely sensitive to size (Takano et al., 2018), material geometry (Rebello Sousa Dias and Leite, 2019), dimension, and dielectric properties (Agrawal et al., 2018) of the surrounding media (Figure 2B). For instance, AuNPs have been produced in different shapes, such as the most common nanospheres, nanoshells, nanorods (NRs), nanostars, nanocages, and core–shell structures (Table 1). Changing the structural geometry of gold nanoparticles can readily and precisely tune the LSPR from visible to the near-infrared (NIR) region without giving up linewidth (Tan et al., 2018). As an important factor that may affect LSPR, the size of gold nanoparticles is multiple. The increased rod aspect ratios of gold nanorods (AuNRs) show a significant peak shift to the near-infrared region (He et al., 2020). Distinctly from AuNRs, gold nanoshells (AuNSs) are highly sensitive to the shell thicknesses, which similarly change their plasmonic properties (Ogunyankin et al., 2018). Going beyond the conventional impact of LSPR factors—the shape and size of GNPs, new synthetic control methods are also the affecting factor of LSPR and require expanding the freedom in structural and functional designs. Yuhua Feng’s group synthetically controlled the non-wetting growth of homometallic nanostructure in two new dimensions (the number and emerging shapes of the Au islands), obtaining a series of Au-on-AuNR hybrid structures, and effectively and continuously tuned their LSPR within visible-NIR spectral range, making them excellent candidates for photothermal therapy and photoacoustic imaging agents (Jia J. et al., 2020). The electric field of LSPR is also highly dependent on the dielectric property of the supporting substrates because the interaction between the gold nanostructures and the surrounding medium may change the refractive index of the GNPs (Bhalla et al., 2019), and when the local refractive index of the NPs increases, this always causes the decreases in LSPR frequency; thus, the sensitivity of the LSPR to the refractive index of the surrounding substrates can also be used for detecting dynamic gold nanostructures and the ambient chemical reactions (Ogunyankin et al., 2018) in the changes in LSPR scattering.

**TABLE 1:** Summary of various gold nanostructures.

**Nanostructure**	**Synthesis methods**	**Characterization**	**Shortcomings**	**References**
Gold nanotube	Template synthesis mechanism	Tunable plasmonic resonances in NIR region; larger active areas and more electroactive sites for immobilizing biomolecules; high scattering contrast; high sensitivity	Cytotoxic potential due to chemical synthesis	Liu et al., 2020
Gold nanorod	Template synthesis mechanism; seed-mediated protocols; seedless methods; the green synthesis	Two typical LSPR peaks with tunable plasmonic resonances in NIR region; high sensitivity; easy surface functionalization; antimicrobial activity mode; strong magnitude of saturable absorption	Cytotoxicity, accumulation in liver *in vivo*, poor photostability.	Alkilany et al., 2012; Ma et al., 2017
Gold nanocluster	Chemical etching; Chemical reduction; Sonochemical synthesis; Electrochemical synthesis; Microwave-assisted synthesis	Tunable plasmonic resonances in NIR region; ultra-small size; strong photoluminescence; sturdy photostability; fast renal elimination; excellent biocompatibility; extremely large surface area	Cell apoptosis	Zhang Q. et al., 2018; Zhao et al., 2014
Gold nanostar	Seed-mediated method; seedless synthesis method; chemical reduction of gold salt method; surfactant-free method	Multiple plasmon resonances; low toxicity; high antibacterial activity; great surface plasmon resonance; easy surface functionalization	Cytotoxicity	Mousavi et al., 2020
Gold nanocage	Galvanic replacement reaction	Enhanced absorption in NIR regions; hollow interiors and porous walls; extraordinarily large scattering and absorption cross sections; easy surface functionalization	Melting point at a power density	Xia et al., 2011
Gold nanoshell	Gold nanoshells on SiO_2_ core: surfactant-assisted seeding method; single step deposition-precipitation (DP) seeding; sandwiched gold seeded shell Gold nanoshells on a polymer core: solvent-assisted approach; combined swelling-heteroaggregation; gold colloid seeding Hollow gold nanoshells: sacrificial template method	Tunable plasmonic resonances in NIR regions; highly effective for PTT and SERS	Weak non-linear response	Ma et al., 2017
Gold nanosphere	Wet chemistry method; seed-mediated growth	Single LSPR peak; the smallest specific surface area; high cellular internalization; highest colloidal stability; easy and available synthetic methods; easy surface functionalization	Cytotoxicity, accumulation in different organs *in vivo*	Wozniak et al., 2017

*LSPR, localized surface plasmon resonance; NIR, near-infrared region; SERS, surface-enhanced Raman spectroscopy; PTT, photothermal therapy.*

**TABLE 2:** The applications of gold nanostructure in tumor environment.

**Main affections on TME**	**Composite**	**Therapeutic modality of GNPs**	**Wavelength**	**References**
	Au@BSA-NHA	CT imaging contrast		Shi et al., 2016
Hypoxia-sensitive nanoprobe	Anaerobic bacteria–GNP conjugates	Photothermal therapy	NIR-II	Luo et al., 2016
Alleviating hypoxia	Au-hemoglobin loaded platelet	Radiosensitizers	NIR-II	You et al., 2020
	ICG-PtMGs(Pt-MOF@GNSs)@HGd	Photothermal therapy CT and MRI imaging	NIR-II	You et al., 2020
	Gold cube-in-cube core	Photothermal therapy	NIR-I	Zhang et al., 2019
Glutathione-responsive drug delivery	SiO_2_@Au@MnO_2_–DOX/Apt	Photothermal therapy	UV-vis spectroscopy and NIR-I	Zhang T. T. et al., 2018
pH-activatable nanoprobe	GNPs-CKL-FA	CT contrast agent	NIR-I	Tang et al., 2019
pH-responsive drug delivery	Gold nanostar@ZIF-8	Drug carriers photothermal	NIR-II	Deng et al., 2019
Cathepsin B-responsive nanomedicine	Multifunctional peptide-coated ultrasmall gold nanoparticles	Radio-sensitizing cytotoxicity	X-ray	Ding et al., 2020
Inactivation of cancer-associated fibroblasts (CAF)	Gold nanoparticles	Inhibit CAF		Zhang et al., 2021
SERS analysis of CAF	MoS2-Au	SERS-active probe	NIR-II	Li et al., 2020b
Normalizing tumor vessels	AuNPs-A&C	Drug delivery	Photoacoustic	Xiao et al., 2017

*TME, tumor microenvironment; GNP, gold nanoparticle; NIR, near-infrared region; CT, computed tomography; MRI, magnetic resonance imaging; SERS, surface-enhanced Raman spectroscopy.*

Besides, LSPR is applied in many imaging techniques, such that dark-field microscopy (DFM) and two-photon luminescence (TPL) primarily use the light scattering property of GNPs, while photoacoustic imaging often applies the optical absorption of GNPs. Two-photon luminescence imaging, through scanning a focused laser beam over the target tissues, has been used to visualize distributions of localized surface plasmons in nanoparticles within the NIR region. This TPL is extremely sensitive to the highly enhanced electro-magnetic fields connected with plasmonic excitations in gold nanostructures (Durr et al., 2007). Consequently, the collective oscillations of the electrons in the gold nanoparticles generate strong TPL signal enhancements. In addition, gold nanorods, as appealing TPL imaging substrates, can strongly scatter to detect cancer cells under excitation in the near-infrared region where biological tissues exhibit slight extinction coefficients (Zhang W. et al., 2018). TPL imaging from gold-nanorod-labeled cancer cells could be acquired by using less than 60 times the laser light excitation power required for two-photon auto-fluorescence (TPAF) imaging of unlabeled cancer cells, consistent to three orders of magnitude amplified in an emitted signal for equal excitation intensity (Durr et al., 2007).

### Near-Infrared Region

**FIGURE 3:**
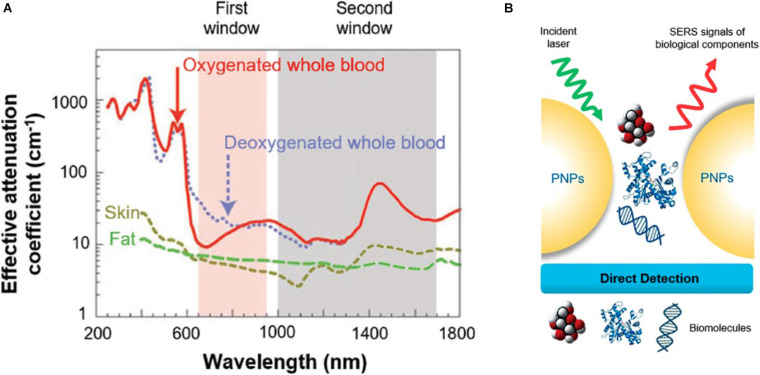
Schematics of **(A)** near-infrared region, **(B)** surface-enhanced Raman spectroscopy. **(A)** The absorption spectrum of biological tissues spanning from 200 to 1,800 nm. The absorption and scattering of whole blood, skin, and fat are significantly reduced in either NIR-I (700–900 nm, shaded in pink) or NIR-II (1,000–1,700 nm, shaded in gray) window. Reprinted with permission from Kenry et al. (2018), Copyright (2018) John Wiley and Sons. **(B)** The direct SERS detection. Reprinted (adapted) with permission from Zong et al. (2018), Copyright (2018) American Chemical Society.

Due to the scattering and absorption of light by biological tissues, UV and visible light own a limited penetration depth into and then propagation out of tissues not taking deep-tissue imaging. Near-infrared light can penetrate more efficiently into tissues such as skin and blood than UV and visible light because the main absorbers of visible and infrared light—water and hemoglobin have their lowest absorption coefficient and scattering of excitation emission photons in the near-infrared region (Shanmugam et al., 2014), thus providing maximum laser penetration in the tissue and minimizing the autofluorescence of the non-target part (Smith et al., 2009). The near-infrared window is divided into two distinct spectral ranges (Figure 3A): NIR-I (traditional NIR window, 700∼900 nm) and NIR-II windows (the second NIR window, 1,000–1,700 nm) (Ji et al., 2020). Chen et al. (2019) synthesized miniaturized gold nanorods which are 5–11 times smaller than the regular size under nanosecond laser irradiation. Small AuNRs absorbing at 1,064 nm, are used to photoacoustic molecular imaging applications that significantly enhanced photoacoustic signal of overall cancer, and lower background noise of endogenous absorbers in tissues. With the advancement of NIR fluorophores and adequate sensitive photon detectors, the NIR window can expand to the longer wavelengths with further reduced scattering, deeper penetration and high-spatial resolution in molecular imaging (Ding et al., 2018). NIR-II fluorescence imaging *in vivo* is a much newer field of research. Compared with traditional imaging approaches using the shorter emission wavelength windows, NIR-II has deeper tissue penetration capability and higher signal–background ratio (SBR). However, due to the poor aqueous solubility, generally low quantum yields, and lack of suitable molecules with bandgaps, the longer fluorescence emission wavelength in small-molecule organic dyes is difficult in the NIR-II window. Fortunately, NIR-I fluorophore indocyanine green (ICG) has been explored for NIR-II imaging of cancer in clinic, and the advantages of NIR-II imaging for tumor imaging and image-guided therapy is worth studying (Zhu et al., 2018). Li et al. (2020a) discovered that Au_25_(SG)_18_ was a highly promising bone-targeted NIR-II probe. Dynamic NIR-II imaging of the whole body in mice receiving Au_25_(SG)_18_ displayed all dorsal bone structures ranging from cranial to spin with high resolution and contrast, and even clearly defined the pelvis and femur (Li et al., 2020a).

### Surface-Enhanced Raman Spectroscopy

The surface-enhanced Raman spectroscopy (SERS) has developed from an esoteric physical phenomenon to an effective analytical measurement technique (Lindquist et al., 2019). Once an incident light hits a sample, the major proportion of the scattered light from the sample have the same frequency, representing the Rayleigh scattering effect. Nonetheless, about a millionth of photons is inelastically scattered from the sample with an energy unlike the incident light energy, corresponding to the Raman scattering effect, which is unique for every molecule providing precise spectral fingerprint of the samples, and the difference between Raman scatter and the incident light is proportional to the molecular vibrational states (Liebel et al., 2020). When molecules are located near a plasmonic material, the Raman signals can be significantly amplificated, leading to the SERS process (Ding et al., 2017; Zong et al., 2018; Figure 3B). There are at least three mechanisms in relation to the enhancement factors: the dominating factor first mentioned is electromagnetic (EM) enhancement induced by the local surface plasmon resonance, which is irradiated by a certain wavelength range light resulting in collective oscillation of the conduction electrons in the nanostructured metal substrate. The second is the chemical enhancement (CM) under resonance related to the charge transfer between the sample molecules and the metal nanostructure substrate, but it is molecule specific, and compared with EM enhancement, it presents a lower enhancement factor. The third possibility is the resonances within the molecule itself (Zong et al., 2018). *In vivo*, Raman signals are inherently weak for imaging and drug location tracking. Surface-enhanced Raman spectroscopy can gain the dramatic amplification of the Raman scattering efficiencies when molecules are grafted on the surfaces of GNPs and take the superiorities in the gold nanostructure plasmon features. After AuNPs efficiently target the cell outer membrane proteins, using SERS from the surface of AuNPs can quantify the local pH induced by H^+^ extrusion in MKN28 gastric cancer cells and HepG2 liver cancer cells, visualizing the dynamics of proton exchange in cells (Puppulin et al., 2018). GNPs coated by target-protein-immobilized substrate in direct SERS-based immunoassay (SERSIA) closely contact target proteins, obtaining the SERS signal of the target protein signal and proving the feasibility of protein imaging at a micrometer range. This detection protein sensitivity was comparable to the ELISA, and the correlation between GNPs and target protein was well matched. Through this method, SERSIA can reliably estimate the limitation of the protein level at a sub-picomolar detection (Shin et al., 2020). As an alternative method to traditional fluorescence-based spectroscopy, SERS not only owns ultrasensitive multiplexing capability (Samanta et al., 2014), non-photobleaching feature (Zhang J. et al., 2018), sub-picomolar level sensitivity, and high signal-to-noise ratio (Amendola, 2019) but also enables ultrasensitive detections of molecular vibrations even at the standard for the high structural selectivity in a single molecule. SERS sensing with a flow-through scheme has been applied in DNA and protein analysis. Gold nanourchins (AuNUs) have many sharp tips. And the electromagnetic field can be concentrated at the sharp tip in the anisotropic structure that can act as an excellent “hot spot,” resulting in a dominant contribution to the SERS intensity. When the SERS platform was utilized to detect a single molecule, nucleotides are mixed in the AuNUs solution to allow an average submonolayer coverage on the surface of the AuNUs. Once trapped, plasmonic hot spots are created by coupling the sidewall of the nanohole and the sharp tips of the AuNUs, which exhibit an intense, confined electromagnetic field for SERS detection of the DNA already adsorbed on the tips. Through applying electric potentials, nucleotides can stay in the hot spots sufficiently long to achieve single-molecule detections (Huang et al., 2019).

### Light-Triggered Modalities–PDT and PTT

#### Photodynamic Therapy

**FIGURE 4:**
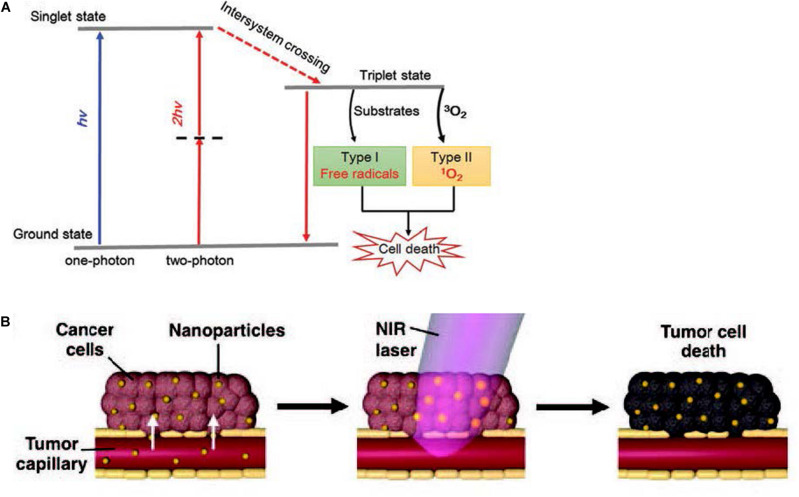
**(A)** Schematic illustration of type I and type II mechanisms in photodynamic therapy (PDT). Reprinted with permission from Shen et al., 2016, Copyright (2016) Royal Society of Chemistry. **(B)** Schematics demonstrating photothermal therapy. Reprinted with permission from Riley and Day (2017), Copyright (2017) John Wiley and Sons.

Photodynamic therapy (PDT) is an emerging and minimally invasive treatment modality with a spatiotemporal selectivity. It employs photosensitizer molecules to absorb the excitation light of a certain wavelength and generate reactive oxygen species (ROS) to selectively damage tumor tissues *in situ*. Three essential factors in PDT are non-ionizing light providing energy for photodynamic reaction, a photosensitizer (PS) harvesting this light energy and participating in the reaction, and molecular oxygen-producing ROS upon electron transfer from excited photosensitizers (Zhou et al., 2016). When the photosensitizer is irradiated to a photon of light, it becomes activated from its ground state (singlet state) to a short-lived excited singlet state and can convert its energy to emit fluorescence or internal transformation into heat, which is the mechanism for quantifying the amount of photosensitizer in cells or tissues and fluorescence imaging *in vivo* monitoring the distribution and pharmacokinetics of the PS. The short-lived excited singlet state PS may also experience the process known as intersystem crossing that the spin of its excited electron inverts to acquire the relatively long-lived (microseconds) excited triplet state (Dolmans et al., 2003). The excited triplet can react with a substrate directly, such as the cell membrane or a molecule, and transfer an electron to form a radical atom. Then these radicals interact with ground-state molecular oxygen to generate oxygenated products including hydroxyl radicals, superoxide anion radicals, hydrogen peroxides, and so on (type I reaction). Alternatively, the energy of the excited triplet can be transferred directly to oxygen, to form singlet oxygen—a highly ROS (type II reaction) (Chatterjee et al., 2008; Figure 4A). Reactive oxygen species (ROS), broadly defined as chemically reactive oxygen-containing small molecules, include the superoxide anion (O_2_^–^), hydroxyl (OH⋅) radicals, hydrogen peroxide (H_2_O_2_), and singlet oxygen (Sies and Jones, 2020). Singlet oxygen is the most prevalent reactive oxygen species produced upon the activation of photosensitizers to induce irreversible damage to tumor cells. Upon extrapolation to an H_2_O-containing cell, a singlet oxygen has a lifetime of ∼3.5 μs (Hatz et al., 2007).

Gold nanoparticles generally display excellent photostability and strong two-photon photoluminescence, owning a high potential to be applied in two-photon excitation (TPE) PDT. The TPE occurs when the sum of the absorption of two lower-energy photons equals the energy of the band gap of the PSs, providing a potential for deeper tissue penetration and lower photo-bleaching of PS molecules (Shen et al., 2016). Furthermore, the TPPL properties of GNPs are strongly correlated with the geometry. The two-photon action cross sections of single gold nanospheres (∼83 GM), nanocubes (∼500 GM), nanotriangles (∼1.5 × 10^3^ GM), nanorod (∼4.2 × 10^4^ GM), and nanobranches (∼4.0 × 10^6^ GM) were dramatically different. Laser-induced melting experiments found that the sharp tips of these nanobranches were closely correlated with observed strong TPPL (Gao et al., 2014).

#### Photothermal Therapy

The vascular system in tumor undergoes agglomeration, distortion, and expansion, resulting in its failure in taking heat away quickly like the normal vascular system does. Owing to inadequate bloodstream and oxygen transport through the newly formed immature tumor vascular system, tumor cells reside in a nutrient-deprived and acidotic milieu that confers them with greater sensitivity to heat (Wang et al., 2016). Meanwhile, malignant cells have impaired heat shock protection mechanisms, so the cytotoxicity profile of mild hyperthermia is usually very good. Temperature rises rapidly above the threshold value of 42–45°C [as biological cells begin to die from hyperthermia when the temperature reaches up to 43°C, on the other hand, high doses of heating (>50°C) induce necrosis resulting in inflammation and is harmful to the neighboring normal cells] for 15–60 min at the intracellular location target sites, leading to ablate cancer cells and consequently to cell death (Figure 4B; Rengan et al., 2015). Conventional methods have poor therapeutic effect on deep-seated tumor, and the heating distribution is always not well controlled, such as hyperthermia produced by microwaves or heat applicators. More importantly, these methods are only macroscopically confirmed to the tumor area but not especially at the cellular level (Vo-Dinh et al., 2013). The investigation of nanoparticles as conduits for generating hyperthermia is tumor focused, minimally invasive, and uniform. Numerous nanomaterials with strong NIR absorption ability present great potential in photothermal treatment, achieving encouraging therapeutic effect *in vivo*.

AuNPs usually exhibit strong absorbance in the NIR region with a much higher absorption cross-section compared with the small organic dyes, whose peak could be changed by the morphology and aggregation state of AuNPs (Goncalves et al., 2020). When the administered gold nanoparticles transmitted in the blood flowed across the tumor vessels under NIR laser irradiation, they convert the effective delivered optical energy into localized heat, causing the tumor temperature to increase (Vo-Dinh et al., 2013; Cheng et al., 2014). The direction of heat loss in GNPs is from inside out reducing damage to normal tissues comparable with other means of external hyperthermia, and they make the steady temperature increase in the tumor (Moy and Tunnell, 2017). Gold nanorods are one of the most studied GNPs in PTT, which have excellent photothermal conversion efficiency and aspect ratio-dependent absorption peak positions. The anisotropic gold nanorod shape splits the LSPR into two plasmon modes (Vigderman et al., 2012): one is a weak short-wavelength band around 520 nm in the visible region formed by the transverse electronic oscillation, which is relatively unaffected by nanorod size; the other is a strong long-wavelength band in the near-infrared region from the oscillation of electrons along the long axis. AuNRs can provide a large electric field, and the enhancements are primarily located in the regions at the two ends of the nanorods (Chen et al., 2013). Combined with various aspect ratios (the ratio between the length and the width), these properties enable GNRs to become attractive candidates for exploitations in PTT.

## Stabilization, Biodistribution, Biocompatibility *in vivo*

Gold nanoparticles can either be directly intravenously injected into the bloodstream or interact with the primary tissue barriers and then translocate into the bloodstream. When GNPs are exposed to blood or biological fluids, proteins are adsorbed onto the GNP surface at first for a few minutes forming a so-called protein corona (PC), which may be well organized by irreversible interactions forming the long-lived hard corona, or through loose “protein–protein” associations forming a rapidly transient layer of biomolecules, providing a soft corona (Feliu et al., 2016). The protein corona cannot only induce steric stabilization and reduce non-specific cellular uptake but also promote the destabilization effect of GNPs by impacting protein-mediated bridging, changing surface charge compensation and inhomogeneity onto the GNPs (Charbgoo et al., 2018). Notably, this kind of proteins forming the outer layer affect the sub-organ biodistribution of AuNPs. Generally, the fate of the GNPs *in vivo* commonly depends on physicochemical properties, mechanical properties, and surface charge. Therefore, this section reviews the mechanical properties of GNPs in blood circulation, biodistribution, and tumor accumulation.

### Stabilization

**FIGURE 5:**
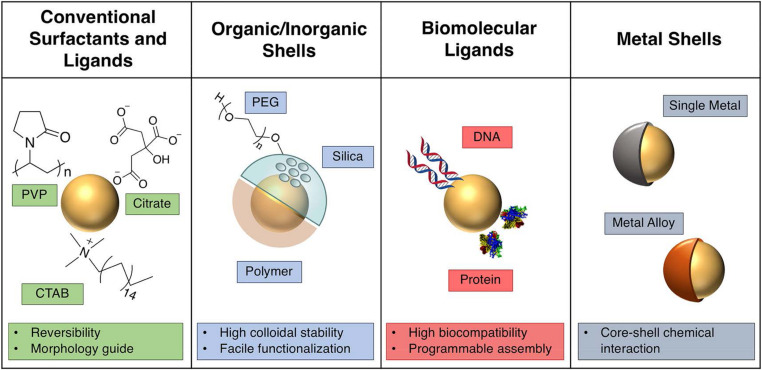
Description of various strategies in colloidal plasmonic nanoparticle. Reprinted with permission from Kang et al. (2019), Copyright (2019) American Chemical Society.

The stabilization of the fabricated gold nanoparticles demonstrating the ability to protect GNPs from aggregation or dissolution in colloidal dispersion is crucial for preservation of the desired plasmonic performance. There are various strategies used to achieve stabilization (Figure 5). The cetyltrimethylammonium bromide (CTAB) as a bilayer via electrostatic interactions absorbed on the gold surface has been predominately utilized as a shape controller and a stabilizer. A bilayer structure around the GNRs consisting of CTAB increases colloidal stability in aqueous solution. CTAB coordinating with another mild reducing agent could obtain anisotropic AuNPs, which is vital for potential applications in SERS. However, its high cytotoxicity is a major concern, and in a biological setting, the CTAB layer must either be exchanged or encapsulated (Shi et al., 2021). Polyethylene glycol (PEG) (Huckaby and Lai, 2018) is another commonly used stabilizer prolonging the blood circulation time *in vivo*. Clinically, PEG is approved for intravenous application (Rafiei and Haddadi, 2017). PEG chains are hydrophilic and non-toxic polymers having a flexible nature, which assemble into dense palisades of tethered chains to achieve unique properties (D’Souza and Shegokar, 2016). The dense PEG shell endows the AuNPs with a stealth character to avoid non-specific recognition by the immune system guaranteeing the biocompatibility of AuNPs’ effective accumulation in the tumor area based on enhanced permeability and retention (Chan et al., 2020). Apart from the optimization of PEG, considering the interaction and the stabilization of GNPs in blood plasma, bovine serum albumin (BSA) as the most abundant plasma protein holds great promise for biomedical applications. The BSA-coated AuNPs showed high colloidal stability and biocompatibility with no hemolytic response, significantly in contrast to CTAB-capped AuNPs (Tebbe et al., 2015). Nevertheless, BSA has been mainly used as a template and a secondary stabilizing agent on account of the structural complexities and difficulties in controlling during modification. Additionally, biomolecules like DNA have been broadly used as a ligand to provide steric stabilization and high electrostatic repulsion forces to GNPs due to strong negative charge on the phosphate backbone (Liu and Liedl, 2018). In general, these strategy stabilities of GNPs are mainly assayed *in vitro*, and firmly grafted stabilizers around gold nanoparticles may degrade when injected *in vivo* leading to the dramatic changes in physicochemical properties and integrity of GNPs (Kreyling et al., 2015). Therefore, future research will likely encompass the enhancement of the stabilities of the AuNPs *in vivo*.

### Blood Circulation

**FIGURE 6:**
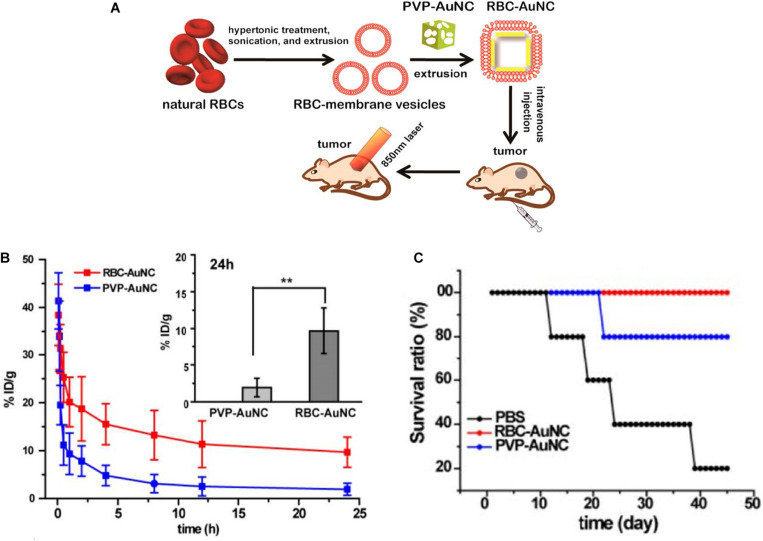
**(A)** Schematic demonstrating the preparation of RBC-AuNCs.**(B)** The RBC-AuNCs retained longer in blood than the pristine PVP-AuNCs in mice. **P < 0.01. **(C)** The survival time of the RBC-AuNCs group was longer than that of PVP-AuNCs and PBS group. Reprinted with permission from Piao et al. (2014), Copyright (2014), American Chemical Society.

Once GNPs are administered into the blood, these nanoparticles are exposed to the dynamic environment of the bloodstream. Flow rate is a fundamental property that influences the transport of gold nanoparticle and interaction with endothelial cells. The decreased uptake of nanoparticles by the endothelial cells occurs in increased flow rates and eventually reduces the amounts of GNPs entering the tumor region through *trans*-endothelial processes (Chen et al., 2020). As mentioned above, despite PEG ensuring the blood circulation of GNPs, the length of the PEG is essential for the circulation times of NPs. Lipka et al. (2010) found that 10-kDa PEG chains have a prolonged blood circulation time than non-PEGylated AuNPs and 750-Da PEG AuNPs. However, PEGylated GNPs suffered from “accelerated blood clearance (ABC) phenomenon” after repeated injection, and consequently, they fail to retain their sustained circulation (Zhao et al., 2017). Interestingly, inspired by the natural long circulation of red blood cells (RBCs), RBC-camouflaged gold AuNCs (RBC-AuNCs) (Figure 6A) exhibited a superior circulation half-life than their biopolymer-stealth-coated counterparts (Figure 6B) and kept good colloidal stability in a mouse model. Moreover, mice treated with RBC-AuNCs achieved 100% survival over a span of 45 days, whereas those receiving PVP-AuNCs or PBS exhibited loss of survival to varying extents (Figure 6C; Piao et al., 2014).

Although little to no dissolution and excretion of large gold nanoparticles (∼10–200 nm) in blood ensures long circulation time, gold’s poor biodegradability induces its impaired physiological clearance *in vivo*. In order to improve this concern for clinical application, ultrasmall gold nanoparticles are designed to be coated with the pH-sensitive polymer and encapsulated within ∼150 nm of polymeric micelle. Micelles were inactive during long blood circulation and showed rapid release from the micelle in acidic buffers (pH 5.0) and in cultured macrophage cell. Mice receiving micelle treatment displayed progressive physiological clearance of GNPs, with the liver elimination > 85% over 3 months (Higbee-Dempsey et al., 2020).

### Passive and Active Targeting

The tumor microenvironment plays a key role in determining passive targeting of GNPs (Figure 1). Tumor vessels defect a well-organized branching hierarchy from large vessels into smaller vessels, and the structure of the vessel wall is abnormal, with an abnormally thick basement membrane, wide interendothelial junctions, a large number of fenestra, and the maximum pore diameters are several hundreds of nanometers (Golombek et al., 2018). Properly formed blood vessels in normal tissue does not allow nanoparticles to pass through because their size is relatively large compared with natural small molecules and growth factors. Therefore, the vascular permeability and hydraulic conductivity of tumors are significantly higher than normal tissues (Yhee et al., 2017). For this reason, after administered intravenously, GNPs easily extravasate through leaky vasculature and accumulate in the tumor. Besides, the lymphatic drainage in tumor tissue is highly impaired and does not drain the fluid efficiently contributing to nanoparticle entrapment and retention. This above phenomenon is often referred to as the enhanced permeability and retention (EPR) effect (Nel et al., 2017).

It is worth noting that inter-endothelial gaps do not account for the transport of nanoparticles into solid tumors, and up to 97% of nanoparticles extravasate into tumors through active trans-endothelial mechanisms (Sindhwani et al., 2020). The term “active targeting” often refers to specific bioconjugation (antibodies or small molecule) grafted to the nanomedicine surface that can specifically recognize tumor and bind to overexpressed receptors with a high affinity in the target region, then mediate nanomedicine–cell interactions, and subsequently enhance therapeutic efficiency (Byrne et al., 2008; Figure 1). These proteins or small biomolecules enable the favorable characteristics of gold nanoparticles to be linked with the biocompatibility, biodistribution, and physical properties (Li L. et al., 2019; Sharifi et al., 2019). Self-assembled monolayers (SAMs) have been applied in modifying the surface of GNPs. SAMs have the possibility to assemble on the surfaces of any shape or size, and they provide a reasonably strong and flexible method to precisely tailor the interfaces between gold nanoparticle structures and molecules in the surrounding environment (gold–sulfur bonds are stable to physiologically salt concentrations and relevant pH) (Schon et al., 2003; Love et al., 2005).

### Biodistribution and Biocompatibility

**FIGURE 7:**
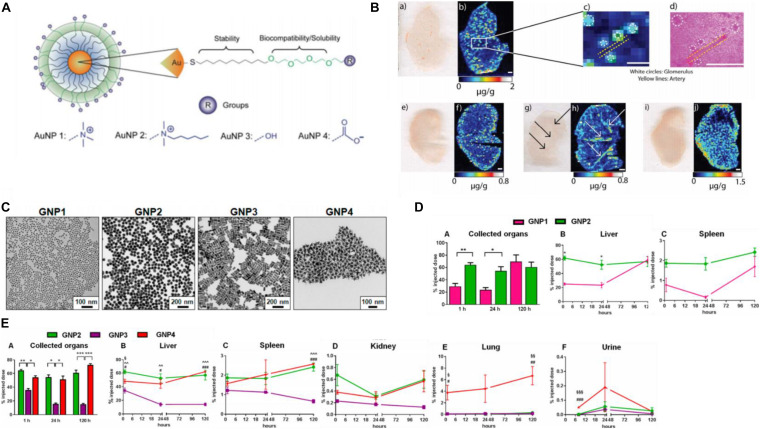
**(A)** Structure of the different surface charge AuNPs. **(B)** Positively charged AuNPs **(a,b,e,f)** accumulated in glomeruli of the kidney tissues **(c,d)**, while neutral ones **(g,h)** were in arteries of the kidney, and negatively charged AuNPs **(i,h)** were more homogeneously distributed. Reprinted with permission from Elci et al. (2016). Copyright (2016), American Chemical Society. **(C)** TEM micrographs of the AuNP library. **(D)** (A) Different sizes influence thekinetics of AuNPs. **(B,C)** The larger size penetrated faster in organs and remained stable a long time. (E) The biodistribution **(A)** of three shapes AuNPs with similar size, spherical, and star-like AuNPs had similar behavior in filter organs **(B–D)** and higher penetration than rod-like AuNPs. Star-like AuNPs showed a high tropism for the lungs **(E)** and higher levels in urine **(F)**. *P < 0.05, **P ≤ 0.005, and ***P ≤ 0.0005 (^∧^GNP2 compared to GNP3, ^§^GNP2 compared to GNP4, and #GNP3 compared to GNP4). Reprinted with permission from Talamini et al. (2017), Copyright (2017), American Chemical Society.

Biodistribution or biodurability is referred as the tendency of NPs to resist dissolution and biotransformation in media or physiologically relevant solutions (Bourquin et al., 2018), controlled by many factors including physicochemical properties (e.g., size and shape), doses administered, administration routes of GNPs, as well as tissue-/blood-dependent permeability. AuNPs do not universally target all cell types. For instance, the surface chemistry of the AuNPs influences the sub-organ biodistributions. Positively charged AuNPs preferentially accumulated in the glomeruli of the kidneys, guarantying that they are rapidly cleared from circulation within 24 h of injection, faster than neutral and negatively charged NPs (Figures 7A,B). Neutral AuNPs had a greater extent of accumulation in the marginal zone with white pulp regions of the spleen and Kupffer cells of the liver, suggesting that these AuNPs extensively interact with the immune system, whereas positively and negatively charged AuNPs did not interact with the immune system as they broadly accumulated in the red pulp of the spleen (Elci et al., 2016). On the other hand, physicochemical parameters are key determinants in biodistribution *in vivo*, greatly affecting the kinetics of excretion and accumulation of AuNPs in filter organs (Figures 7C,D). Spherical and star-like AuNPs showed the same trend of accumulation except in the liver, and their ability to penetrate were higher than the rod-like AuNPs. In addition, star-like AuNPs were exclusively able to accumulate in the lungs (Figure 7E). Very interestingly, changes in the geometry did not influence poor accumulation in the kidneys of the GNPs themselves and the passage in the blood–brain barrier (Talamini et al., 2017).

The mononuclear phagocytic system MPS and renal system compete with the tumor for circulating NPs and eliminate the most of administered nanoparticles. The MPS is mainly composed of organs containing resident phagocytic cells, such as the liver and spleen. In the liver, Kupffer cells can recognize the opsonin adsorbed on the GNPs flowing through the liver sinusoidal capillaries and phagocytize them (Fischer et al., 2010). The macrophages in the spleen can potentially engulf circulating nanoparticles, but the specifics have not been fully investigated. The epithelium lining of the kidney contains filtration slits of 4–6 nm in width (Venkatachalam and Rennke, 1978), and GNPs smaller than this size may be filtered out of the blood and eventually excreted in the urine. Other major organs including the lymph nodes and the skin also have the ability to remove nanoparticles from the bloodstream.

Sufficient biocompatibility to ensure the innate functions of the normal cells are not disrupted or impaired, and is critically important for nanomaterial application *in vivo*. Gold nanoparticles are not biodegradable and would retain inside the body for long periods of time after administration. Despite tremendous potentials of clinical applications, the toxicity of AuNPs remains a controversial and important issue. Various researches consider that some factors such as surface medication, concentration, and physical dimensions (Pan et al., 2007) play an important part in affecting the cytotoxicity of the gold nanostructures. Surface functionalities are the major player in governing biocompatibility of GNPs, which are crucial for their successful implementation into the clinic. Studies indicate that some precursors such as CTAB, which is bound to the gold nanoparticles might be harmful to the cell, but the nanoparticles themselves are non-toxic (Connor et al., 2005; Li et al., 2018). Connor et al. (2005) found that 18-nm gold nanoparticles containing the CTAB displayed significant toxicity similar to the cytotoxicity of CTAB alone. They centrifuged and washed the CTAB-modified nanoparticles with deionized water three times to wipe out free CTAB in order to find whether the CTAB-modified nanoparticles or unbound CTAB induced the observed cytotoxicity. The results revealed, under the conditions examined, that washed CTAB-modified nanoparticles were detected to be not toxic, which suggested that the coating molecules’ cytotoxicity was attributed to the toxicity of the gold nanoparticles (Connor et al., 2005). Fraga et al. (2014) have found that two different modifications with the same hydrodynamic size and surface charge, capped on the AuNPs, had a very similar distribution pattern at each time-point and mainly accumulated in the hepatic and spleen tissues. Through examining the electron-dense deposits and major clinical changes, they proposed that the surface chemistry seems to have more effect on the toxicity rather than on the biodistribution of short term and long term *in vivo* (Fraga et al., 2014). Zhang et al. (2011) found that the 10- and 60-nm PEG-coated AuNPs induced a significant increase in aspartate transaminase and alanine transaminase levels; meanwhile, the decrease in creatinine was observed in mice treated with the 60-nm particles, which indicated the damage to the liver and kidneys. This article also noted that the preference distribution of different size particles may affect the immune system, but even these particles broke down *in vivo*, and they did not cause appreciable toxicity (Zhang et al., 2011). Conversely, Kharazian et al. (2018) used molecular dynamic simulation (MD) to study the interaction of GNPs with fibrinogen (Fg), and their results revealed that the bare gold surface has a critical role in inducing Fg conformational changes regardless of the type or presence of a surface coating. They also reinforced the MD results in ultra-small gold (∼10 nm or smaller). Although the surface coatings either partially degraded in biological media or contained gaps due to disorganization integrity of the surface coverage, NPs still had the capacity to induce the unfolding of Fg. These results potentiate concerns about the unforeseen immunoreaction of GNPs *in vivo* (Kharazian et al., 2018).

Besides, intracellular gold nanorods (GNRs) are recently reported to have perturbed the ionic microenvironment within the nucleus and altered gene expression in human cells. Despite GNRs were localized in the cytoplasmic vesicles with no genomic DNA interaction, intracellular localization could lead to the separation of gold species from the GNR surface into the nucleus, resulting in structural alterations of genomic DNA (Ho et al., 2018). The cytotoxicity of AuNRs in vascular smooth muscle cells (VSMCs) was strongly correlated with the binding forces and the binding probability between the AuNRs and the cell membrane. Poly(diallyldimethyl ammonium chloride-coated AuNRs) (PDDACAuNRs), having a relatively large size binding force and large binding probability, were non-toxic in cancer cells in previous reports, whereas it displayed extreme toxicity to VSMCs and was detrimental to VSMC contractile function. When the rational design of nanoparticles for medical applications are considered, it is necessary to assess the cytotoxicity of gold nanoparticles on cells, as the cytotoxicity of AuNRs is cell specific (Sun et al., 2018).

## Detection for Cancer

### Gold Nanoparticles in Biomarker Detection

Due to the remarkable plasmonic properties and large surface area to volume ratio of gold nanoparticles, they have been widely investigated for their potential applications in biomarker detection. Biomarkers may be molecules or something overexpressed in blood and serum or at the region of cancer that facilitate diagnosis. Detection of some biomarkers occurring in ultralow levels is challenging and require ultrasensitive detection strategies (Viswambari Devi et al., 2015).

**FIGURE 8:**
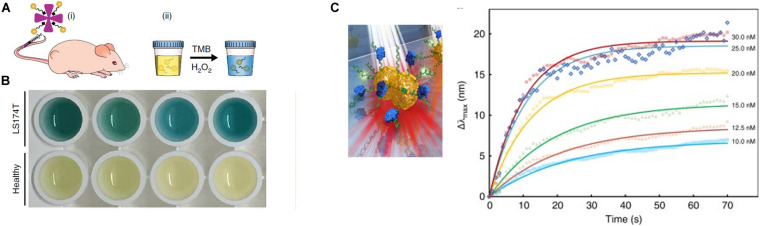
**(A)** Schematic of the renal clearance assay, including two steps (i and ii). **(B)** Colorimetric assay of urine from tumor and healthy-bearing mice injected with AuNC-P220-Nav. The blue color urine is belonging to samples from tumor-bearing mice. Reprinted with permission from Loynachan et al. (2019), Copyright (2019), Springer Nature. **(C)** Illustrations of single NP sensing (sNPS) models and real-time monitoring of MutS binding to each point mutation and a homoduplex DNA, non-specific sequence binding of MutS to point mutations change LSPR signals. Error bars represent mean ± SEM. Reprinted with permission from Ma et al. (2019).

Nanozymes have shown a broad range of applications in *in vitro* detection (Zhu et al., 2019). GNPs as a nanozyme material, compared with platinum and gold–platinum bimetallic hybrid nanoclusters, possesses the highest catalytic activity and low coefficient of production variation (8.5%), and can be designed as a scalable excellent diagnostic platform. The smaller-sized nanomaterials exposing higher surface-to-volume ratio and more active sites have a better catalytic activity (Wu et al., 2019). Ultrasmall (<2 nm) AuNCs mastering intrinsic catalytic activity and efficient renal clearance properties, with the presence of surface peptide modification, are able to monitor a diverse range of diseases in the urine (Zhang et al., 2015). After exposure to complex physiological environments, such as patient serum and urine, AuNCs still retained a high stability and effectively evaded non-specific protein adsorption. Neutravidin (NAV), which have low non-specific binding and high affinity for biotin properties (Jain et al., 2017), coupled to the catalytic AuNCs, forming MMP-responsive nanosensors, specifically cleaved MMP or the serine protease thrombin. In tumor-bearing mice, AuNC–NAV complexes could be actively disassembled by elevated MMP levels at the site of the disease, and then proteolytically liberated AuNCs, by virtue of their small size, were efficiently filtered into the urine to be analyzed by TMB catalytic activity assay and ICP–MS (Figures 8A,B). This system can be developed to diagnose a range of diseases in microenvironments (Loynachan et al., 2019).

Moreover, gold nanoparticles can detect genetic mutations in tumor. The single NP-sensing (sNPS) technology is a promising approach to achieve non-invasive assays for elucidating subtle variations in genes (Li L. et al., 2019). The sensing scale of LSPR in gold nanoparticles can be reduced to a single NP, which owns a high-throughput signal-to-noise ratio (S/N). The refractive index of single NP relies on the structure and localized sensing volume/area of the NP. Gold-bridged NPs as a signal source under low-energy white light showed twofold higher sensitivity than gold nanorods. Furthermore, the ionic environment of the DNA interface can alter Au-bridged NPs. When mutation sequence non-specifically bind to point mutations, LSPR signals change and different point mutations own variable reactions (Figure 8C). Through the sNPS system and the typical atlas, this product sensors can effectively and directly identify subtle distortions in biomolecular binding and genetic alterations (Ma et al., 2019).

Gold nanoparticles can act as SERS hotspots to detect cancer (Wang Z. et al., 2018). Volatile organic compounds (VOCs), serving as biomarkers of the non-invasive recognition of lung malignancies, are always difficult to detect for the reason that high velocity of VOCs makes a low absorptivity on solid substrates and weak Raman scattering. The ZIF-8 layer, providing a porous character, was coated onto a self-assembly of gold superparticles (GSPs) to form a GSPs@ZIF-8 SERS substrate, could affect the traveled fashion and process of gaseous molecules, and induce high-intensity electromagnetic fields. Through Schiff base reactions, the pregrafted amino group of 4-ATP in GSPs@ZIF-8 sensitively captured gaseous aldehydes (released at tumor-specific tissue) at the ppb (parts per billion) level, providing a promising opportunity for *in vitro* diagnoses of early stage lung cancer (Qiao et al., 2018).

### Imaging

#### Photoacoustic Imaging

**FIGURE 9:**
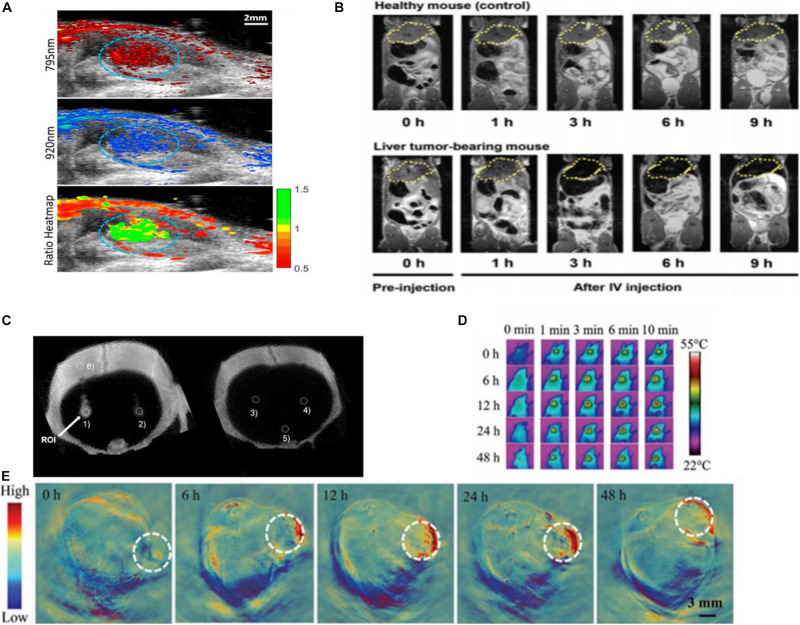
**(A)** The US/PA image of labeled stem cells (circled in blue) *in vivo*. Reprinted with permission from Dhada et al. (2019), Copyright (2019), American Chemical Society. **(B)** MRI imaging using SPAuNC (aff+) as a contrast agent from the mice bearing a liver tumor and healthy mice; the tumor-grafted liver was clearly differentiated from the normal liver of healthy mice. Reprinted with permission from Kwon et al. (2017), Copyright (2017), John Wiley and Sons. **(C)** *In vivo* micro-CT images of AuNP-PLL-RITC-labeled hMSCs. Reprinted with permission from Kim et al. (2017), Copyright (2017) John Wiley and Sons. **(D)** Photothermal and **(E)** photoacoustic imaging of Capan-1 tumor-bearing nude mice injected with rGADA. Reprinted (adapted) with permission from Jia X. et al. (2020), Copyright (2020) John Wiley and Sons.

Photoacoustic (PA) imaging, is a non-invasive imaging modality, obtaining real-time information based on converting absorbed short-pulsed laser energy into ultrasound through the thermoelastic effect. These ultrasound waves can be detected by ultrasound transducers at the tissue surface that converts the mechanical acoustic waves to electric signals and after duly processing to form an image representing the absorbed optical energy (Fu et al., 2019). Photoacoustic imaging has better tissue contrast related to the optical properties of different tissues, without the limitations in the mechanical properties of biological tissues existing in ultrasound imaging. AuNPs possess a high molar extinction coefficient, which can maximize the amount of light absorbed, optimal tissue penetration to avoid the strong absorption of intrinsic chromophores, and are not susceptible to photobleaching (Qiu et al., 2020). PAI choosing AuNPs as exogenous contrast agents can indicate the presence of various tumors by monitoring the passive or active accumulation of receptors which are functionalized on the AuNPs in the tumor, or map sentinel lymph nodes to trace the metastasis of cancer cells (Dumani et al., 2019), and provide a specific target in tracking intravascular PA (IVPA) imaging of macrophages in atherosclerotic plaques (Hui et al., 2017). PA imaging can track stem cells based on gold nanoparticles as a contrast agent. GNPs have good loading into stem cells and are not appreciably exocytosed (Figure 9A). The contrast agent system consists of inert gold nanorods bound with ROS-sensitive near-infrared dye IR775c (stem cells produce ROS to degrade the cell). This nanoplatform can track cell viability in real time with high spatial and temporal resolution, and assess the contribution and efficacy of cell therapies (Dhada et al., 2019). Besides, AuNPs encapsulated within the lipid shell of microbubbles (MBs) can act as a US-responsive PA imaging probe for *in vivo* “background-free” PA imaging. AuNPs separated by the lipid shell of MBs showed relatively low NIR PA signals. Whereas Au@lip MBs were exposed to US pulses, they would burst into nanoscale aggregates of Au@lip NPs and exhibit significantly enhanced NIR photoacoustic signals due to their red-shifted surface plasmon resonance absorbance. The tissue background PA signals could be realized by subtracting the PA image captured pre-US burst from that after US burst, enabling background-free PA imaging with high sensitivity. As a proof of concept, Au@lip MBs as a multimodal probe for US-responsive background-free PA imaging is a particularly promising strategy for detection of changes in tumor blood perfusion after drug treatment (Meng et al., 2019).

#### Magnetic Resonance Imaging

Magnetic resonance imaging (MRI), as the widely used diagnostic modalities in the clinic, is non-ionizing, non-invasive, and radiation free. Under an external magnetic field, magnetic nuclei align to allow resonance through a radiofrequency pulse (Zhou et al., 2019). The resolution of MRI for disease diagnosis can be affected by using MRI contrast agents that improve the contrast by shortening the longitudinal (T_1_) or transverse (T_2_) relaxation times of protons (Jeon et al., 2020). Although superparamagnetic iron oxide nanoparticles (SPION) were used to be applied as contrast agents of T_2_-weighted MRI, clinics no longer allow SPIONs *in vivo* because of their ROS toxicity causing severe cellular damage and inflammatory responses (Kwon et al., 2017). The GNPs with anisotropic geometries and shapes elevate local field inhomogeneity under an external magnetic field (Yang et al., 2019). The glutathione (GSH)-responsive Au nanowreath (AuNW) effectively guided magnetic imaging (Figure 9B). Exceedingly small magnetic iron oxide nanoparticles (ESMIONs) decorated on AuNWs make this nanostructure not merely effectively quenched with the T_1_-weighted MRI due to the strong T_2_ decaying effect but also responded to glutathione (GSH) and had the T_1_ imaging function “turning on.” After intravenous injection, the relatively high GSH concentration in tumor microenvironment induced the switch of T_1_ signal of magnetic AuNWs from an initially “OFF” state to “ON” state. The larger assemblies of ES-MIONs enhanced their tumor accumulation compared with ES-MIONs alone, providing higher MRI contrast imaging (Kwon et al., 2017).

#### Computed Tomography

Another commonly employed non-invasive clinical imaging technique is computed tomography (CT), which relies on the X-ray attenuation by the tissues (Lee et al., 2013). AuNPs are promising CT contrast agent materials because gold has a high atomic number and density, thus, possessing an intrinsic high X-ray absorption coefficient (Sabale et al., 2017). Citrate-stabilized GNPs coated with poly-L-lysine (PLL) and rhodamine B isothiocyanate (RITC) enable CT cell tracking *in vitro* and *in vivo* (Figure 9C; Kim et al., 2017) and may provide applications in CT image-guided interventions utilized in the injection of cellular therapeutics.

#### Photothermal Imaging

The photothermal effect can be detected by a thermal imaging system to form photothermal imaging (PTI). PTI requires a heating beam and a probe beam to detect refractive index variations induced by their increase in the local environment. Its high sensitivity of detection is at the single-particle level in a dark background. Gold nanoparticles are ideal probes for PTI due to their biocompatibility, fast relaxation times, and large absorption sections around plasmon resonance (Willets et al., 2017). Wang et al. introduced rGO@AuNSDODAB/DOPE-FA (rGADA) for photoacoustic/photothermal dual-modal imaging-guided photothermal and gene synergistic therapy of pancreatic cancer. From photoacoustic imaging in Capan-1 tumor-bearing mice, the facilitated accumulation of the rGADA in tumors displayed considerably distinct photoacoustic signals (Figure 9E). At the same time, the PTI showed that the temperature of tumor increased promptly to above 55°C (Figure 9D), which was relatively high enough for the ablation of the tumor. These results suggested that the combination of reduced graphene oxide (GO) and AuNS improved the dispersity and photothermal efficiency (Jia X. et al., 2020).

## Gold Nanoparticles in Therapy

### Drug Delivery

In cancer chemotherapy, anticancer drug resistance can be intrinsic (primary) or acquired after repeated cycles of chemotherapy (secondary). Chemotherapeutic drugs fail to activate or alter drug-appointed targets and enhanced drug efflux from the drug accumulation regions, are the established mechanisms that markedly lower anticancer efficiencies in tumors (Lepeltier et al., 2020). In the current researches of cancer medicine, significant efforts have been devoted to explore smart nanoparticles for an effective targeted drug delivery system (TDDS) (Luther et al., 2020). In designing nanomedicines, there are several points to be considered, such as preferential concentration in the tumor tissue, efficient internalization into cancer cells, and accurate subcellular location of drugs at their site of actions. GNPs can be utilized as complex systems, including nanocarrier-mediated combination therapies maximizing the therapeutic efficacy and NIR light-induced drug-responsive release to overcome mechanisms of drug resistance (Sharifi et al., 2019). Although tumor microenvironment heterogeneities cause many obstacles to successful GNP delivery such as the clearance of immune cells and reduced permeation, researchers designed the nanomaterial drug delivery systems based on the characteristics of the tumor microenvironment to enhance therapeutic efficacy and accuracy.

Due to hypoxia in the tumor microenvironment and glycolysis as the main strategy for glucose metabolism, the cancer cells are trapped in acidic extracellular micromilieu (McDonald et al., 2019). An effective anticancer pH-sensitive nanosystem should have the ability to store and stabilize the drug at physiological pH, rapidly release the payloads at the trigger point pH, and ensure a therapeutic dose of drug concentration at the intracellular level. There are usually three strategies for pH-responsive drug release in the tumor microenvironment: the first approach is to utilize chemical groups wherein the changes in physical or chemical properties depend on pH and accept or donate protons; the second approach is to apply acid-labile chemical covalent bonds between drug molecules and the surfaces of nanocarriers, or to construct new nanocarriers (Liu et al., 2014); and the third method is the pH-responsive “PEG detachment”(Kanamala et al., 2016).

The acidic conditions of the tumor microenvironment (TME) inspire tumor immunotherapeutic efficacy. Jibin Song’s group developed dual pH/GSH-responsive nanogapped gold nanoparticle vesicles carrying an immune inhibitor (BLZ-945) and an anticancer polymeric prodrug for synergistic concurrent chemo-immunotherapy. The vesicles were fabricated by self-assembly of amphiphilic GNPs grafted with hydrophilic PEG and dual pH and redox-response copolymerized prodrug PSN38VP [poly (SN38-co-4-vinylpyridine)], which contained the reduction-responsive disulfide (−S-S−) bond and pH-responsive 4-vinylpridine (4-VP). The theranostic nanoplatform also can guide cargo release by showing photoacoustic (PA) intense PA signal in the NIR-II window. The responsive AuNNP@SN38/BLZ-945 released nearly 90% of BLZ-945 at pH 6.5, whereas negligible leakage of BLZ-945 was detected at pH 7.4, suggesting that AuNNP@SN38/BLZ-945 could selectively release BLZ-945 at tumor pH and maintain stability in neutral pH. Following dissociation of the pH-responsive nanovesicles, the PA signal intensity decreased due to attenuation of the plasmonic coupling between GNPs and the “blue-shift” of absorbance. The characteristic of GSH-responsive released SN38 in the reductive microenvironment exerted cytotoxic effects. Therefore, GNPs as nanocarrier and imaging agents, combined with pH/GSH-responsive, have a superior nanostructure in inhibiting the growth of tumor (Zhu et al., 2020).

In subcellular acidification compartments, a nanoparticle-directed drug can be delivered to the endosomes and lysosomes for pH-responsive intracellular release (Liu et al., 2014). The pH-responsive system can utilize oligodeoxynucleotides (ODNs) packed on the nanoparticles to modulate the hybridization stability controlling the assembly and disassembly of nanoparticles in neutral or acidic pH, and its duplex structure can be utilized to load intercalative drugs. Based on the features mentioned above, Won Jong Kim and his workers designed the cytosine (C)-rich i-motif sequence to link sAuNPs for clustering into size-tunable large clusters (cAuNPs), and i-motif sequence was added into iBO-sAuNPs at neutral pH, causing the formation of cluster. In an acidic pH, the cytosine (C)-rich i-motif formed a unique tetrameric structure by partial hybridization of C and protonated C. Following cellular uptake, the i-motif and iBO-sAuNPs dissociated at acidic endosomal pH. Taken together, this dynamic DNA nanocluster provided higher tumor specificity and efficient drug release (Kim et al., 2014).

Hamishehkar’s group developed a new type of smart core/shell of gold NPs decorated by a pH-responsive copolymer of thiolated polyethylene glycol-b-poly to target and trigger the delivery of MTX. Amino and thiol side groups in the backbone of a copolymer consisting of a polymeric shell were used to couple a polymer and a load of MTX to the surface of gold NPs. Moreover, due to the folate-mediated endocytosis behavior of MTX, this developed NPs were valued for their potential in efficient cell uptake. Under the acidic condition of a simulated cancer tissue (pH 5.3, 40°C), MTX was released three times faster than the physiological state (pH7.4, 37°C), and 78% of the MTX was released from the nanocarriers. Nevertheless, only 26% of the loaded MTX was released in physiological conditions (pH 7.4, 37°C) after 48 h. These results suggest that polymers with the ionizable functional groups grafted on the GNPs could be promising pH-responsive systems (Ghorbani and Hamishehkar, 2017).

### Photodynamic Therapy

The hypoxic tumor microenvironment limits the ROS generation of PDT due to oxygen deficiency (Zhou et al., 2016). Increased cellular metabolism, mitochondrial dysfunction, and oncogene activity promote the excessive accumulation of ROS in cancer cells. The major source of endogenous ROS is continually produced in the mitochondria. Electron leakage from the electron transport chain in the mitochondria, primarily in the reaction process mediated by coenzyme Q (CoQ), ubiquinone, and its complexes, may react with molecular oxygen to release mitochondrial superoxides, which can subsequently be converted to other ROS through chemical reactions. Overproduction of ROS can oxide cellular components, destruct the mitochondria, and disturb normal metabolism leading to cytotoxicity. Unfortunately, although the ROS level in TME is relatively high compared with that of the normal tissues, the amount of endogenous ROS is insufficient to stimulate rapid release of large quantities of monomeric drugs (Zhang L. et al., 2018). The ROS-responsive systems combining the PDT would serve as a powerful exemplary approach to overcome the aforementioned drawbacks. The hyaluronidase-responsive size-reducible biomimetic nanoparticles (mCAuNCs@HA) was a synergetic combination therapy for chemotherapy, PDT, and immunotherapy. In this study, the photosensitizer pheophorbide A (PheoA) and ROS-responsive prodrug PXTK were co-loaded to facilitate drug release. The synergetic effect of PDT-induced immunogenic apoptosis and PD-L1 blockade induced immunosuppressive boosted host immune system. The combination therapy provided extraordinary tumor inhibition and antitumor immune response (Yu et al., 2019).

Gold nanoparticles can act as a vehicle to carry a high payload of photosensitizers, forming the GNR–photosensitizer complex. The complex using the target tissue receptors or antigens modified on the surface of GNR passively localize on the diseased site. With the PS released from the GNR, the NIR fluorescence imaging detect the tumor with a high signal-to-background ratio, and the tumor can be selectively destroyed in a non-invasive manner with minimizing phototoxic damage of the surrounding normal tissues in photodynamic theranostic (Gamaleia and Shton, 2015). Besides, the presence of gold nanoparticles in the complex improved the quantum yield of singlet oxygen production significantly. Instead of acting as a transport carrier activating PSs in the vicinity, gold nanoparticles without the presence of any organic photosensitizers, can directly sensitize the generation of singlet oxygen through irradiation with light. Recently, AuNCs, composed of several to hundreds of gold atoms and generally < 2 nm in diameter (Higaki et al., 2019; Zhou et al., 2019), have multiple peaks in their ultraviolet-visible absorption spectra and discrete electronic energy levels, which sharply distinguish them from AuNPs (Gao et al., 2019). AuNCs emerge as a promising ideal PS candidate for PDT. For the reasons that AuNCs are chemically pure and have eco-friendly synthesis (Poderys et al., 2020), they exhibit excellent biocompatibility and low toxicity, long tumor retention and rapid normal tissue clearance, and the microsecond (μs) range fluorescence lifetime is conducive to the generation of excess ROS. Dihydrolipoic acid-coated gold nanocluster (AuNC@DHLA) possessed superior two-photon optical properties and strong ROS generation when stimulated by an external laser, and showed highly efficient antitumor effects in a hepatocellular carcinoma xenograft model with negligible toxicity (Han et al., 2020).

### Photothermal Therapy

Despite advances in nanoparticle design of heating trials, most photothermal studies failed to take into account the effect of the tumor environment on light-to-heat conversion efficiency. Claire Wilhelm and his workers systematically studied the light-to-heat conversion efficiency of gold nanostars from aqueous dispersion to cancer cells cultured *in vitro* and injected to solid tumors *in vivo*. Their results elucidated that the heating efficiency of gold nanostars in aqueous dispersion was governed by particle size and laser wavelength, while the heat generation of nanostars internalized by cancer cells *in vitro* still appeared very efficient, but was size and laser independent. Meanwhile, the heat generation of nanostars intratumorally injected *in vivo* evolved with time in cellular internalization. Together, the common recorded photothermal conversion efficiencies in aqueous dispersion cannot predict the values acquired from either isolated cells or living tumor tissues, and the most important design features of gold nanostars for thermotherapy were their size and coating affecting optimal biodistribution *in vivo* rather than their plasmonic peak measured in aqueous dispersion (Espinosa et al., 2016).

## Conclusion and Outlook

With the rapid development of nanomedicine, various organic or inorganic nanoparticles have been investigated, among which gold nanoparticles have attracted increasing attention due to its feasible functionalized surface, excellent tumor specificity, high drug-loading capacity, and biocompatibility. Multifunctional designs of gold nanoparticles enabling the *in situ* imaging such as computed tomography (CT) and MRI, or chemotherapy drug delivery and the anti-cancer effects of PDT and PTT, have been constructed. In this review, we discussed the optical and plasmon properties of gold nanoparticles, then elucidated their physical principle in detail. The physicochemical properties of GNPs support them to provide unique and sensitive nanoplatforms for imaging, diagnostic, and therapy. Of these GNP characteristics, localized surface plasmon resonance as the essential and powerful optical property is used through surface-enhanced processes including SERS, surface-enhanced fluorescence, and other plasmonic performances (Mejia-Salazar and Oliveira, 2018).

We briefly summarized the current applications of GNPs and introduced the collected researches of GNPs on theranostic systems in recent years, particularly emphasizing on these functional GNPs applications in TME. Before entering the tumor region, the GNPs experience the process of blood circulation and biodistribution. We demonstrated the series of biological behaviors *in vivo* and discuss the nanotoxicity of GNPs. Tumor environment is an indispensable field for anti-cancer nanotechnology researches, breaking away from which to do cancer treatment studies is meaningless. GNPs have been engineered to response or profit from particular aspects of the TME to acquire selective targeting of the tumor region, or an environment-responsive therapeutic cargo, and precisely accumulate at the appointed regions. However, such high-performance nanoplatforms, applied in the complex tumor microenvironment, remain intractably challenging. For example, except the well-known hypoxic microenvironment, the tumor microenvironment is also characterized by hypertonic characteristics. The elevated intertumoral penetrating pressure in the extracellular matrix (ECM) surrounding cancer cells can dramatically affect plasmonic properties of the GNPs that bring the quite variant optimal therapeutic properties between in model media and *in vivo* (Espinosa et al., 2016). Nevertheless, few researches focus on the hypertonic tumor media in the solid tumors. Additionally, the dense extracellular matrix (glycoproteins, proteoglycans, hyaluronic acid, etc.) and fibrotic tissue, which varies with tumor types, always hamper nanoparticles’ access to targeted tumoral regions and limit their further penetration (Danhier, 2016). Moreover, GNP-based nanomedicines are still in preclinical stage with only few translations from the laboratory studies to clinical trials. More works are required to simulate the actual scenes of occurrence in tumor and elucidate the mechanisms associated with its applications in medicine. Overall, it is essential to explore the possibilities of gold nanoparticles in TME applications and make more theoretical breakthroughs in order to promote the process of nanotechnology applied in more clinical practice.

## Author Contributions

QG: writing—concept. JG, ZZ, and HZ: original draft preparation and discussion. JZ: artwork. QG and DW: review and editing. All authors: contributed to the article and approved the submitted version.

## Conflict of Interest

The authors declare that the research was conducted in the absence of any commercial or financial relationships that could be construed as a potential conflict of interest. The handling editor declared a past co-authorship with one of the authors, HZ
